# Dorsal-Ventral Differences in Retinal Structure in the Pigmented Royal College of Surgeons Model of Retinal Degeneration

**DOI:** 10.3389/fncel.2020.553708

**Published:** 2021-01-18

**Authors:** Una Greferath, Mario Huynh, Andrew Ian Jobling, Kirstan Anne Vessey, Gene Venables, Denver Surrao, Helen Christine O'Neill, Ioannis J. Limnios, Erica Lucy Fletcher

**Affiliations:** ^1^Department of Anatomy and Neuroscience, The University of Melbourne, Parkville, VIC, Australia; ^2^Clem Jones Centre for Regenerative Medicine, Faculty of Health Sciences and Medicine, Bond University, Gold Coast, QLD, Australia

**Keywords:** retina, photoreceptor, retinitis pigmentosa, Müller cell, microglia, outer limiting membrane

## Abstract

Retinitis pigmentosa is a family of inherited retinal degenerations associated with gradual loss of photoreceptors, that ultimately leads to irreversible vision loss. The Royal College of Surgeon's (RCS) rat carries a recessive mutation affecting mer proto-oncogene tyrosine kinase (merTK), that models autosomal recessive disease. The aim of this study was to understand the glial, microglial, and photoreceptor changes that occur in different retinal locations with advancing disease. Pigmented RCS rats (RCS-p^+/^LAV) and age-matched isogenic control rdy (RCS-rdy ^+^p^+^/LAV) rats aged postnatal day 18 to 6 months were evaluated for *in vivo* retinal structure and function using optical coherence tomography and electroretinography. Retinal tissues were assessed using high resolution immunohistochemistry to evaluate changes in photoreceptors, glia and microglia in the dorsal, and ventral retina. Photoreceptor dysfunction and death occurred from 1 month of age. There was a striking difference in loss of photoreceptors between the dorsal and ventral retina, with a greater number of photoreceptors surviving in the dorsal retina, despite being adjacent a layer of photoreceptor debris within the subretinal space. Loss of photoreceptors in the ventral retina was associated with fragmentation of the outer limiting membrane, extension of glial processes into the subretinal space that was accompanied by possible adhesion and migration of mononuclear phagocytes in the subretinal space. Overall, these findings highlight that breakdown of the outer limiting membrane could play an important role in exacerbating photoreceptor loss in the ventral retina. Our results also highlight the value of using the RCS rat to model sectorial retinitis pigmentosa, a disease known to predominantly effect the inferior retina.

## Introduction

Retinitis pigmentosa (RP) refers to a family of inherited disorders characterized by gradual loss of rod, followed by cone photoreceptors (Hartong et al., 2006). Over 150 causative mutations have been identified affecting the expression of a range of rod-specific proteins or proteins important for retinal pigment epithelial function. The mer proto-oncogene receptor tyrosine kinase (merTK) is known to be involved in phagocytosis of photoreceptor outer segments by the retinal pigment epithelium. Up to 2.5% of cases of autosomal recessive RP are attributed to mutations in merTK (Gal et al., 2000; Oishi et al., 2014). The Royal College of Surgeon's (RCS) rat is a well-established model of inherited retinal degeneration arising from a deletion of 409 base pairs in the gene encoding merTK (D'Cruz et al., 2000; Nandrot et al., 2000). In response to an anomaly in photoreceptor outer segment phagocytosis, there is gradual build-up of outer-segment debris from postnatal day 18 (Dowling and Sidman, 1962; Fletcher and Kalloniatis, 1996, 1997). The accumulation of debris within the subretinal space is thought to be critical to the subsequent photoreceptor loss that ensues. Indeed, by ~2 months of age, retinal function and photoreceptor number is substantially reduced (Adachi et al., 2016; Ryals et al., 2017).

The RCS rat has been extensively utilized for the preclinical development of a range of therapeutic treatments targeting retinal degeneration. Subretinal injection of viral vectors, stem cell therapies or therapeutic agents have been investigated with measurement of the outer nuclear layer thickness as the primary outcome (Conlon et al., 2013; Ghazi et al., 2016; Thomas et al., 2016; McGill et al., 2017; Surrao et al., 2017). In order to accurately assess these and other potential treatments using the RCS rat model, a full understanding on the temporal and spatial changes that occur in the retina with age is required. Notably, variability in retinal structure and function in those with retinitis pigmentosa (RP) has been well-described (Bird et al., 1988). Although it is well-known that the mid-peripheral retina is affected in the early stages of disease, those with autosomal RP associated with rhodopsin mutation display at least two distinct phenotypic subtypes, with some patients displaying difference in the extent of photoreceptor loss in the superior compared to inferior retina (Aleman et al., 2008; Jacobson et al., 2016). Very little is known about the temporal and spatial changes that occur in the retina of rodent models of retinal degeneration, including the RCS rat retina, especially of the pigmented strain of RCS rats. Vascular anomalies affecting regions of the retina close to the optic nerve from 12 weeks of age and that extended inferiorly with age have been reported, implying that photoreceptor loss may also vary with retinal eccentricity or location (Zambarakji et al., 2006; Shen et al., 2014). Indeed, the rate of cone inner segment loss in the albino RCS rats is greater in the inferior and temporal retina than the superior retina (Huang et al., 2011). In contrast, other reports using optical coherence tomography did not identify differences in photoreceptor loss with eccentricity or retinal location (Adachi et al., 2016; Ryals et al., 2017).

Eye pigmentation is another factor that may influence the rate of degeneration. Lowe et al. (2019) showed that pigmentation reduced the rate of photoreceptor loss in the Pro23His (line 3) rat model compared to rats on an albino background. In contrast, pigmentation had no influence on the S334ter model of inherited retinal degeneration (Lowe et al., 2019). Although changes in retinal integrity have been extensively studied in the albino RCS rat strain (Dowling and Sidman, 1962; LaVail, 1981; Adachi et al., 2016; Tan et al., 2020), changes in the structure and function of the pigmented RCS strain is poorly understood.

The central aim of this study was to characterize the changes in retinal structure and function in the pigmented strain of RCS rat, so as to determine whether photoreceptor loss occurs in a uniform pattern across the retina. Our data shows that the ventral retina is affected prior to the dorsal retina and that the presence of an intact outer limiting membrane appears to be an important factor correlated with photoreceptor integrity. These results will be important in the design and interpretation of therapeutic interventions in the RCS rat model.

## Materials and Methods

### Animals

All experimental procedures using animals were performed in accordance with The University of Melbourne Animal Experimentation Ethics Committee (Ethics number: 1312958) and with the recommendations of the Association for Research in Vision and Ophthalmology (ARVO) statement for the Use of Animals in Ophthalmic and Vision Research.

Dystrophic pigmented RCS (RCS-p^+^/LAV) rats (*n* = 74) (referred to here as RCS) and age-matched non-dystrophic control (RCS- rdy ^+^p^+^/LAV) rats (*n* = 71) (referred to here as rdy) aged postnatal day 18, 1, 2, 4, 6, and 12 months were examined (*n* = 8–19 per age per strain). Rats were housed at a controlled temperature (22 ± 1°C) on a 12 h light: 12 h dark cycle and at lux levels varying from 3 to 23 lux. Food and water were available *ad libitum*.

### Electroretinogram Recording

Rats were dark adapted overnight and anesthetized via a mixture of ketamine (60 mg/kg, Provet, Victoria, Australia) and xylazine (5 mg/kg, Provet). The corneal reflex was anesthetized via topical administration of Alcaine (0.5%, Alcon Laboratories, Victoria, Australia), while pupil dilation was achieved via Atropine (1%, Aspen Pharmacare, NSW, Australia) and phenylephrine hydrochloride (10%, Bausch and Lomb, NSW, Australia). Sterile saline (0.9%, Aero Healthcare, NSW, Australia) was applied to the cornea to prevent dehydration. Rats were placed on a heat pad to maintain body temperature while under anesthesia.

ERG recordings involved placing an active electrode on the cornea and a reference electrode under the tongue of the rat. The electrodes were made from silver/silver-chloride wire (Ag/AgCl). ERGs were recorded by generation of a 2.1-log cds/m^2^ full field flash from a Nikon camera (Nikon SB900; Nikon, Lidcombe, NSW, Australia) delivered from a custom made Ganzfeld. Responses were amplified (gain × 5,000, −3 dB at 1 Hz, and 1 kHz; ADInstruments Pty.Ltd, Castle Hill, NSW, Australia) and digitized at 10 kHz. Two consecutive flashes, with an interval of 0.8 s, elicited a mixed rod and cone pathway response and a cone-only pathway response, respectively (Phipps et al., 2007; Jobling et al., 2013; Vessey et al., 2015). Measurements were taken in triplicate and then averaged. To obtain the rod response, the cone response was subtracted from the mixed response. ERG recordings were managed using Scope software (version 3.6.10, ADInstruments Pty.Ltd) on an Apple Macintosh computer (Apple, Inc., Cupertino, California, USA).

Since the responses obtained from dystrophic RCS rats were small, it was not possible to model the ERG responses. Instead, raw ERG data were utilized. The a-wave was defined as the amplitude of the baseline to the trough of the negative slope preceding the leading positive slope. The b-wave was defined as the trough of the a-wave to the peak of the leading positive slope. If no a-wave was present, the b-wave was measured from the pre-stimulus baseline to the peak of the leading slope. The implicit timing of the a- and b-waves were measured from the time of flash to the a-wave trough or b-wave peak, respectively (Jobling et al., 2015).

### *In vivo* Retinal Imaging Using Optical Coherence Tomography

Optical Coherence Tomography (OCT) and fundus images were taken on a Micron III rodent fundus camera (Phoenix Research Labs, California, USA) using specialty Micron III (Phoenix Research Labs) and Streampix software (Norpix Inc., Quebec Canada). Both RCS and rdy animals were anesthetized as above and then imaged following completion of ERG recording. Throughout imaging, lubricating eye gel (Genteal 0.3%, Alcon Laboratories, Victoria, Australia) was applied and the pupil further dilated with atropine when required.

OCT images that were generated from scans across the retina within two disc diameters of the optic nerve. For quantification of retinal layers, an annular scan centered on the middle of the optic nerve head was used to generate OCT images that were of similar retinal eccentricity. The thickness of individual retinal layers was achieved using a custom Image J script (NIH), that segmented each raw B-san (Schneider et al., 2012). Changes in total retinal thickness, outer nuclear layer, inner nuclear layer, inner plexiform layer, and ganglion cell layer were quantified. In addition, the thickness of debris was quantified by measuring the layer bounded by the RPE and outer plexiform layer. Assessment of regional differences in the inner and outer retina were made by measuring the ratio of the dorsal and ventral retina in each animal taken from the annular scan.

### Tissue Collection and Processing

Anaesthetized rats were euthanized by intracardial injection of lethabarb (Virbac Pty.Ltd., NSW, Australia) immediately prior to tissue collection. For orientation, the dorsal region of the eye was marked with a tattoo pen, eyes were removed, the anterior components dissected, and the posterior eye cup placed in 4% paraformaldehyde (PFA) in 0.1 M phosphate buffer (PB) for 30 min. The eye cups were subsequently cryoprotected in graded sucrose solutions (10, 20, 30% v/w) and snap frozen and stored at −80°C until use.

Frozen sections (14 μm) were cut on a cryostat (Hyrax C60, Zeiss, Germany), mounted onto poly-L-lysine coated glass slides (Polysine® Adhesion slides, Thermo Scientific, VIC, Australia) and stored at −80°C until use. All sections were cut from the dorsal to ventral region of the eye, as close to the optic disc as possible, to allow for consistency between eyes.

### Immunohistochemical Labeling and Analysis

In order to evaluate how photoreceptors, microglia and retinal Müller cells change with increasing degrees of degeneration, vertical sections of the retina were processed for indirect immunofluorescence using protocols previously described (Puthussery and Fletcher, 2004; Vessey et al., 2014; Greferath et al., 2016). Briefly, frozen sections were incubated with either a single or a combination of primary antibodies diluted in antibody buffer (3% v/v normal goat serum, 1% w/v bovine serum albumin, 0.05% w/v sodium azide, 0.5% v/v Triton-X in PB). The primary antibodies used in this study included: two markers of gliosis rabbit anti-Glial fribrillary acidic protein, (1:10,000; Cat. No# Z0334; DAKO, Carpinteria, CA, USA) and mouse anti-Nestin (ms anti-Nestin, 1:100; Cat. No# MAB353; Millipore, Frenchs Forest, NSW, Australia,), a marker for either short or medium/long wavelength cone opsins, (rabbit anti-human S or M/L opsins, 1:100,000; kind gifts from Dr J. Nathans), marker for cones, Peanut Agglutinin-Rhodamine (Vector Labs; Cat. No# RL-1072, diluted 1:250); a marker for microglia and macrophages, rabbit anti-Iba1 (Wako Pure Chemical Industries, VA, USA, Cat. No# 019-19741, 1:1500), a marker for Müller glial cells, mouse anti-glutamine synthetase (mouse anti-GS, clone GS-6, Cat. No# MAB302; Merck-Millipore, Frenchs Forest, NSW, Australia, 1:1000), a marker for rod photoreceptor opsin, rhodopsin (mouse anti-rhodopsin, clone 1D4, 1:200, LifeSpan Biosciences, Seattle, WA, USA), an antibody for tight junctions and the outer limiting membrane (OLM), mouse anti-Zonula Occludens-1 (1:200, ZO-1; Immunogen, human recombinant ZO-1 fusion protein encompassing amino acids 334-634, Clone # ZO1-1A12, Cat. No# 339100, Molecular Probes, Life Technologies, Mulgrave, VIC, Australia) and an antibody for vesicular cell adhesion molecule (monoclonal rabbit anti-vCAM1, Cat. No# ab134047, Abcam, Melbourne, VIC, Australia, 1:500), a rabbit anti-CD3, (Abcam Cat. No#ab5690 1:100), an anti-CD8a (Cat. No 10070, 1:100 Biolegend Pty Ltd San Diego, USA), an anti-mouse CD192 (CCR2; Cat. No 150609 1:100 Biolegend Pty Ltd San Diego, USA). For some antibodies (anti-ZO-1, anti-vCAM1) antigen retrieval was required and frozen sections were boiled in 0.1 M citrate buffer (pH 6) for 3 min. Although the antigen retrieval process can impact the integrity of frozen sections, we used at least 20 sections per slide ensuring that there were enough sections for quantitative analysis. Following antigen retrieval sections were washed and incubated in primary antisera overnight. Sections were washed and incubated with appropriate Alexa Fluor® conjugated secondary antibodies (1:400, Life Technologies, Mulgrave VIC, Australia) together with the nuclear stain BisBenzimide H (Höchst, 0.1 mg/ml, Sigma Alderich; Castle Hill, NSW, Australia, Cat. No# 14530) for 1 h. Finally, the sections were washed, cover-slipped with fluorescent mounting media (DAKO, Carpinteria, CA, USA) and imaged using a confocal microscope (Zeiss LSM800, Germany). Images were edited using LSM Zeiss software, ImageJ or CorelDRAW (CorelDRAW, Graphics Suite 2017).

For 3D reconstruction of microglial and Müller cell- contacts, vertical cryostat sections were imaged as Z- stacks and reconstructed using IMARIS software (x64, v.7.7.0 Bitplane AG, Zürich, Switzerland). Confocal z-stacks were reconstructed into 3D images in the *Surpass* mode. Background noise of each fluorescent channel was minimized individually on the *Display adjustments* panel. Images were cropped to the relevant part of the field without altering the resolution. 3D representations of GS-positive Müller cells touching Iba1-positive microglia in the subretinal space in confocal z-stacks were created with the *Surface* function. Rendered images of different magnifications were captured using the *Snapshot* function and exported as Tiff files.

The area and thickness of subretinal debris was measured in confocal images of vertical cryostat sections of dorsal, intermediate and ventral retina of 2 month old RCS rats (at least three sections per eye, *n* = 5 RCS eyes) using the area measurement tool in Image J. In parallel the coverage of GS positive Müller cell sprouting into the debris area was measured the same way and compared.

### Determination of Cell Death

Cell death was detected using a terminal deoxynucleotidyl transferase biotin-dUTP nick end labeling (TUNEL) detection kit (DeadEnd Flurometric TUNEL System, #TB235, Promega Corporation, WI, USA) as previously described (Downie et al., 2010). Vertical cryostat sections were washed in 0.85% sodium chloride for 5 min, rinsed in phosphate buffered saline (PBS) for 5 min, and then incubated in 0.2% Triton-X-100 in 0.1 M PB for 12 min. The retinal sections were then washed three times in PBS for 5 min and equilibrated (200 mM potassium cacodylate; pH 6.6; 25 mM Tris-HCL, 0.2 mM DTT, 0.25 mg/ml BSA, 2.5 mM cobalt chloride) for 10 min. Sections were covered in a solution containing equilibration buffer, nucleotide mix (50 μM fluorescience-12-dUTP, 100 μM dATP, 10 mM Tris-HCl, 1 mM EDTA, and rTdT enzyme for 1 h at 37°C. The reaction was stopped with a buffer containing 0.3 M sodium chloride and 0.15 M sodium citrate at pH 7.2 and sections were subsequently rinsed three times in PBS for 5 min. After TUNEL labeling, cell nuclei were counterstained with BisBenzimide H and coverslipped in mounting medium. TUNEL stained slides were imaged on the Zeiss LSM800 (Zeiss, Oberkochen, Germany). Apoptotic cells were counted using Cell Counter on Image J. Cells were counted from images taken at 20x magnification. For this, sections through the optic nerve (ON) were taken and the region extending from the ON to the ciliary body was divided into five equal parts. Counts were done in central (the region closest to the optic nerve head), mid-peripheral (middle two regions) and peripheral retina (that region closest to ciliary body). All images for analysis were adjusted to the same brightness and contrast. The number of apoptotic cells was counted and expressed per millimeter of retinal length.

### Statistical Analysis

Statistical Analysis was carried out in Graphpad Prism 9 (San Diego, California, USA) and data expressed as the Mean ± Standard Error of the Mean (SEM) unless otherwise stated. One or Two way ANOVA was used where comparisons across two factors or more means were evaluated and a Tukey's *post-hoc* test was used to make individual comparisons as appropriate (Graphpad Prism version 9; GraphPad Software, Inc.). In all figures, statistical significance is expressed as ^*^p < 0.05, ^**^p < 0.01, ^***^p < 0.001, and ^****^p < 0.0001.

## Results

The central aim of this study was to evaluate how retinal structure and function in the pigmented RCS rat retina changed with both age and location in comparison to age matched congenic control rdy retinae.

### Retinal Structure and Function Are Reduced by 1 Month of Age

**Figure 1:**
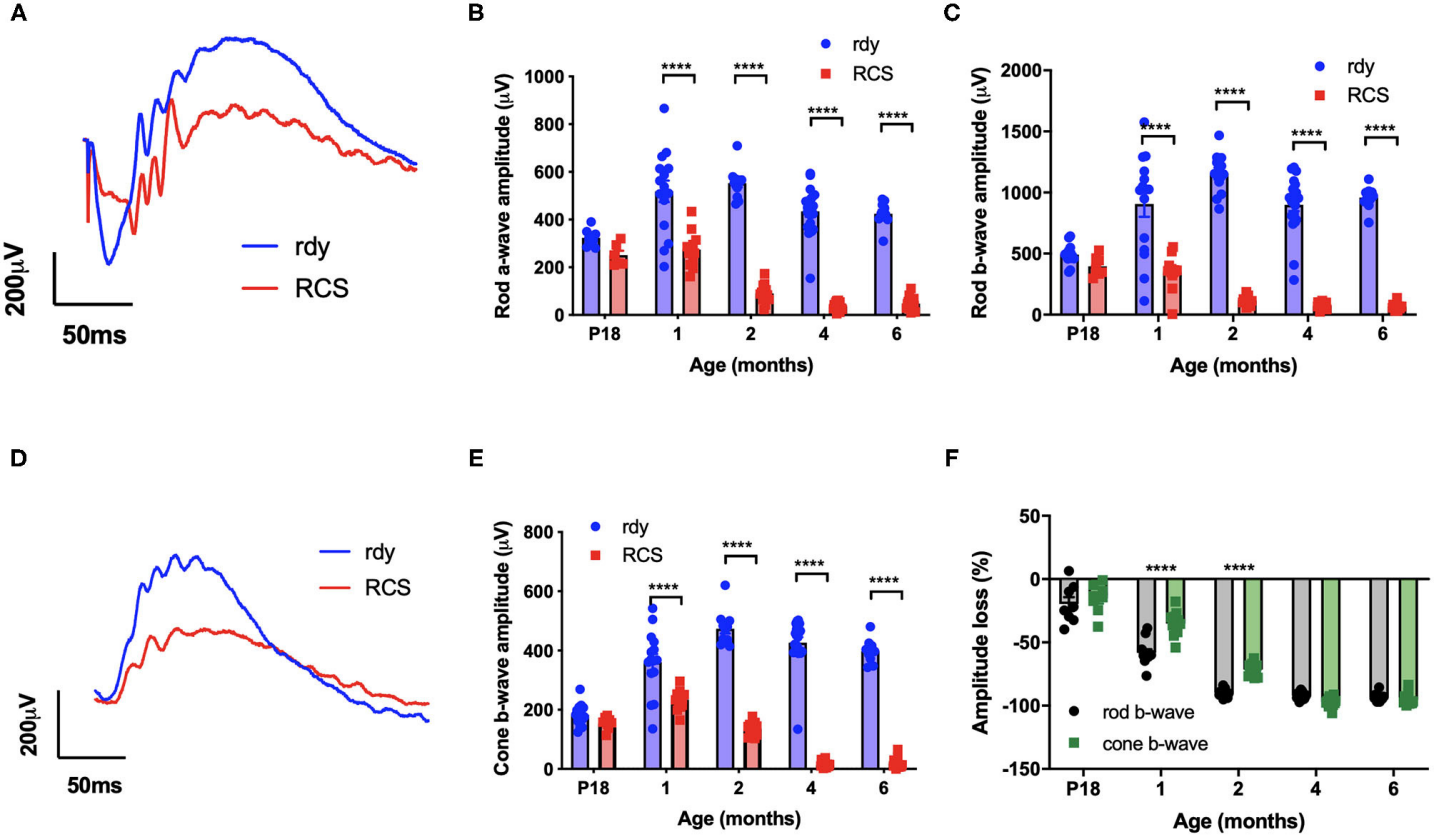
Retinal function at different ages in RCS vs. rdy control rats. **(A,D)** Representative mixed (rod/cone mediated) **(A)** and cone mediated **(D)** ERG waveforms recorded from rdy (gray) and RCS (black) rats aged 1 month. **(B,C,E)** Graphs showing the mean ± SEM of the **(B)** rod a-wave amplitude, **(C)** rod b-wave amplitude and **(E)** cone b-wave amplitude of rdy (control, clear bars) and RCS (filled bars) rats aged postnatal day 18 to 6 months. Inner and outer retinal function significantly reduced from 1 month of age. **(F)** Graph showing the mean ± SEM percentage loss of rod (gray bar) and cone (filled bar) b-wave in the RCS rat aged postnatal day 18 to 6 months of age. Percentage change was derived by comparison with rdy control function at different ages. The loss of the rod b-wave was greater than the cone b-wave at 1 and 2 months of age. (*n* = 10–20 rdy rats per age; 6–18 RCS rats per age; Two way ANOVA, *post-hoc* Tukey's test; ****p < 0.0001).

We first evaluated the time course of retinal functional changes that occur with age using the flash electroretinogram (ERG). Rod and cone mediated function was evaluated by quantifying function in response to a paired-flash stimulus in animals aged postnatal day 18, and 1, 2, 4, and 6 months of age (*n* = 10–20 rdy rats and 6–18 RCS rats per age) (Phipps et al., 2004). Representative rod and cone-mixed waveforms from 1 month old animals are shown (Figures 1A,D). Retinal function was significantly attenuated in RCS rats by 1 month of age as shown in Figures 1B,C,E (Two way ANOVA, strain *p* < 0.0001; age *p* < 0.0001; interaction *p* < 0.0001; *post-hoc* Tukey's test; *p* < 0.0001 for comparisons at 1, 2, 4, and 6 months). In particular, the amplitude of a- (B), b- (C), and cone-b (E) waves steadily declined from 1 month of age and was almost undetectable by 6 months of age. The electroretinogram is a serial waveform, and therefore the observed reduction in rod b-wave could be attributed to losses in photoreceptor or alternatively changes in inner retinal function. We compared the percentage loss of the rod a-wave with the rod b wave and found the losses in each waveform to be similar across the different ages (P18: 22 vs. 19%; 1 month: 47 vs. 61%; 2 month 83 vs. 89%; 4 month: 91 vs. 92%), suggesting that the loss in b-wave amplitude could be explained by photoreceptor dysfunction. Finally, we compared the extent of loss in cone mediated and rod mediated function by assessing the relative loss of the cone b- wave with that of the rod b-wave (Figure 1F). There was a significantly greater loss in rod b-wave than cone b-wave in animals aged 1 and 2 months of age, suggesting that rod mediated pathways were affected to a greater extent than cone mediated pathways (Two way ANOVA with *post-hoc* Tukey's test; *p* < 0.00001 for comparisons at 1 and 2 months of age).

**Figure 2:**
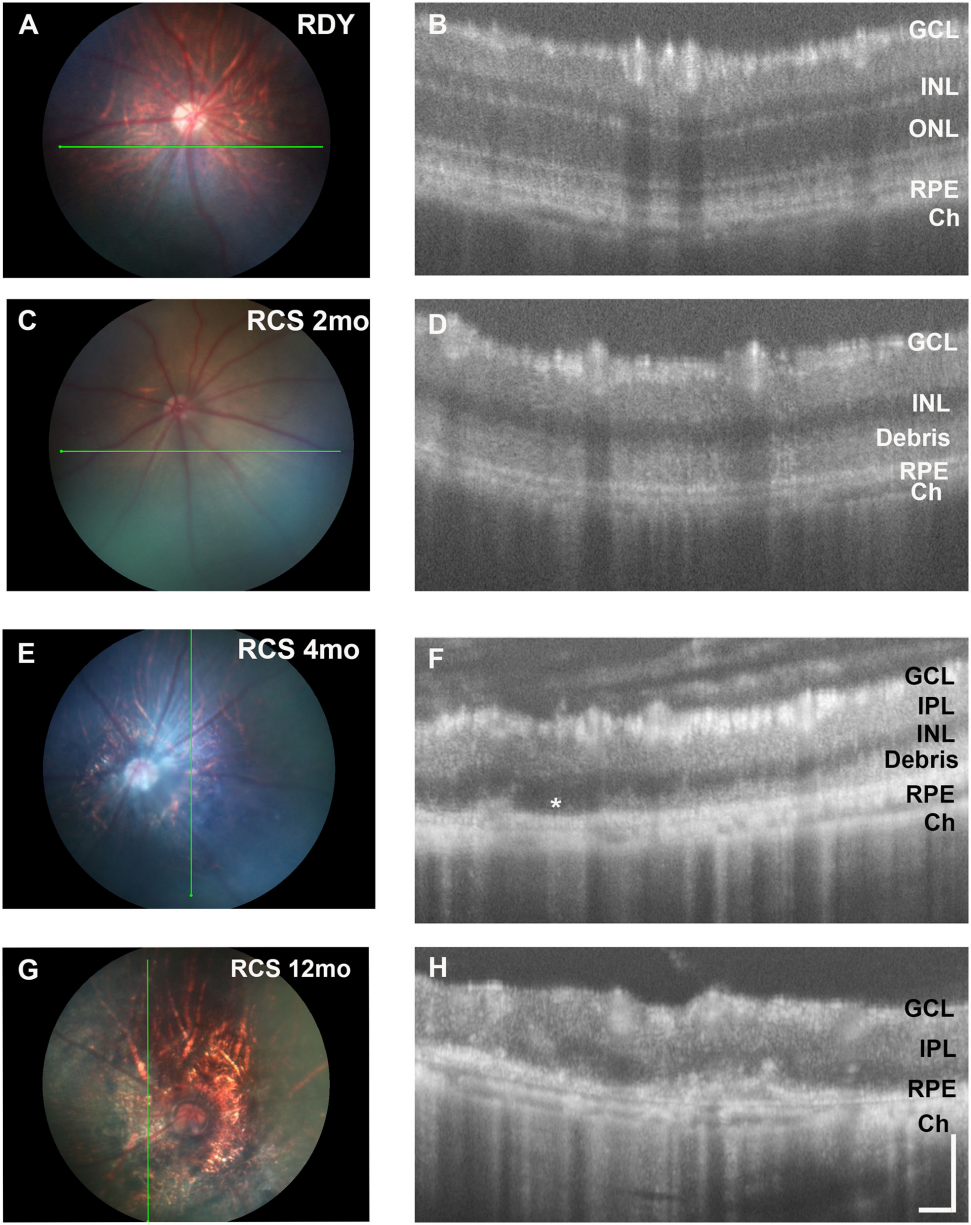
Retinal structure of the RCS and rdy control retinae. **(A,B)** Fundus images of the **(A)** 2 month old rdy rat and RCS rats aged **(C)** 2 months, **(E)** 4 months, and **(G)** 12 months, with corresponding OCT images shown in **(B,D,F,H)**. In contrast to the rdy rat retina, an area of hyperreflective material is evident in the distal 2 months old RCS retina **(D)**. This layer of debris was found to be patchy and of variable thickness from 4 months of age **(F)** and by 12 months of age **(H)**, the RCS rat retina was extremely thin, with areas of distortion. Asterisk in **(F)** indicates an area where no debris is visible, in contrast to an area immediately adjacent. ONL, outer nuclear layer; INL, inner nuclear layer; IPL, inner plexiform layer; GCL, ganglion cell layer; RPE, retinal pigment epithelium; Ch, choroid Scale bar vertical−100 μm; horizontal−50 μm.

Next, we evaluated changes in retinal structure with age using fundus imaging and optical coherence tomography (OCT). Figure 2 shows representative fundus images of a control rdy rat aged 2 months (A,B) and then RCS rat aged 2 (C, D), 4 (E,F), and 12 (G,H) months. In contrast to the rdy retinal fundus, the retinal structure of the RCS appeared pale potentially because of the accumulation of debris within the subretinal space. Representative OCT images of the rdy and RCS rat at 2, 4, and 12 months are shown in Figures 2D,F,H, respectively. Of note was the variation in the extent of debris accumulation within the subretinal space at 4 months of age (asterisk in Figure 2F).

**Figure 3:**
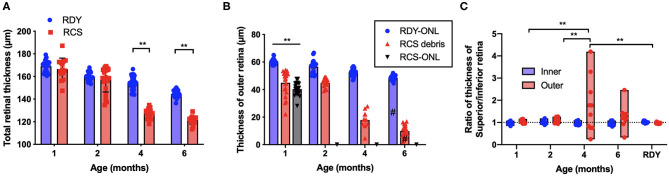
**(A)** Graph of mean ± SEM thickness of the total retina of rdy (blue) and RCS (red) rats aged 1–6 months (*n* > 20 rdy and *n* > 15 RCS rats per age). Total retinal thickness was reduced in the RCS rat at 4–6 months of age (Two way ANOVA *post-hoc* Tukeys test, *p* < 0.0001 for comparisons at 4–6 months). **(B)** Graph of mean ± SEM thickness of the outer nuclear layer of rdy (rdy-ONL; blue bars) and RCS rats (RCS-ONL; black bars) as well as the layer of hyperreflective material (debris; RCS-debris; red bars) within the subretinal space of the RCS rat aged 1–6 months. Although there was a reduction in outer nuclear layer on the rdy rat with age (Two way ANOVA, *post-hoc* Tukey's test ^#^*p* < 0.0001 for comparison between 1 and 6 months), the outer nuclear layer in the RCS rat was significantly thinner than the rdy by 1 months of age, and undetectable at 2, 4, and 6 months of age. In contrast, a significant layer of hyperreflective debris was present in the RCS rat at all ages, but that showed a decline at 4 and a further decline at 6 months of age (Two way ANOVA, *post-hoc* Tukeys test, **p < 0.001 for comparisons at 1 month; *p* < 0.0001 for comparisons of debris between 2 and 4 months and 2 and 6 months; ^#^*p* = 0.0031 for comparison between 4 and 6 months). **(C)** Graph showing the ratio of the thickness between the dorsal (superior) and ventral (Inferior) inner and outer retina. Of note was the variability in this ratio in RCS rats aged 4 and 6 months, with many animals showing a thicker outer retina dorsally than ventrally. (One way ANOVA, *post-hoc* Tukeys, **p < 0.001).

Segmentation analysis was used to quantify changes in total retinal thickness and thickness of the inner and outer retina rdy and RCS retinae with advancing age (*n* = 10–20 rdy and *n* = 10–15 RCS rats per age; (Figure 3). Annular OCT scans were used for this purpose so as to ensure quantification occurs at the same eccentricity in all quadrants of the retina. Total retinal thickness was reduced with age and rat strain, particularly at 4 and 6 months of age (Figure 3A; Two way ANOVA, *post-hoc* Tukey's test *p* < 0.001 for comparisons at 4 and 6 months). As shown in Figure 3B, thickness of the outer retina, which includes the ONL and subretinal debris, changed with age. Although there was a small reduction in outer nuclear layer thickness in the control rdy retina with age (Two way ANOVA, *post-hoc* Tukey's test *p* < 0.001 for comparisons of 1 and 6 months), there was considerable thinning of the outer nuclear layer in the RCS by 1 month of age and virtually no outer nuclear layer present at 2, 4 or 6 months of age (Two way ANOVA, *post-hoc* Tukeys test; *p* < 0.0001 for comparison between rdy and RCS outer nuclear layer at 1 months). Instead, a thick layer of hyperreflective material or debris could be measured from 1 month of age in the RCS rat (Figure 3B). The layer of debris thinned in the RCS rat retina by 4 months of age, with further thinning by 6 months of age (Two way ANOVA, *post-hoc* Tukeys test; *p* < 0.0001 for comparison between 2 and 4 and 2 and 6 months; *p* = 0.0031 for comparison between 4 and 6 months). The decrease in debris thickness corresponded to the reduction in total retinal thickness observed at 4 and 6 months. A comparison of the thickness of the inner and outer retina in the dorsal (superior) and ventral (inferior) retina is shown in Figure 3C. Note that the outer retina is defined here as any photoreceptor nuclei within the outer nuclear layer as well as debris in the subretinal space. In the rdy rat, the thickness of both the inner and outer retina is similar in the dorsal and ventral retina (resulting in a ratio close to one). In contrast, there was considerable variability in the thickness of the outer retina in the dorsal retina in RCS rats aged 4 and 6 months, with some animals showing considerably thicker outer retina in the dorsal retina than ventral retina. It should be noted that these differences were evident in the central region of retina.

**Figure 4:**
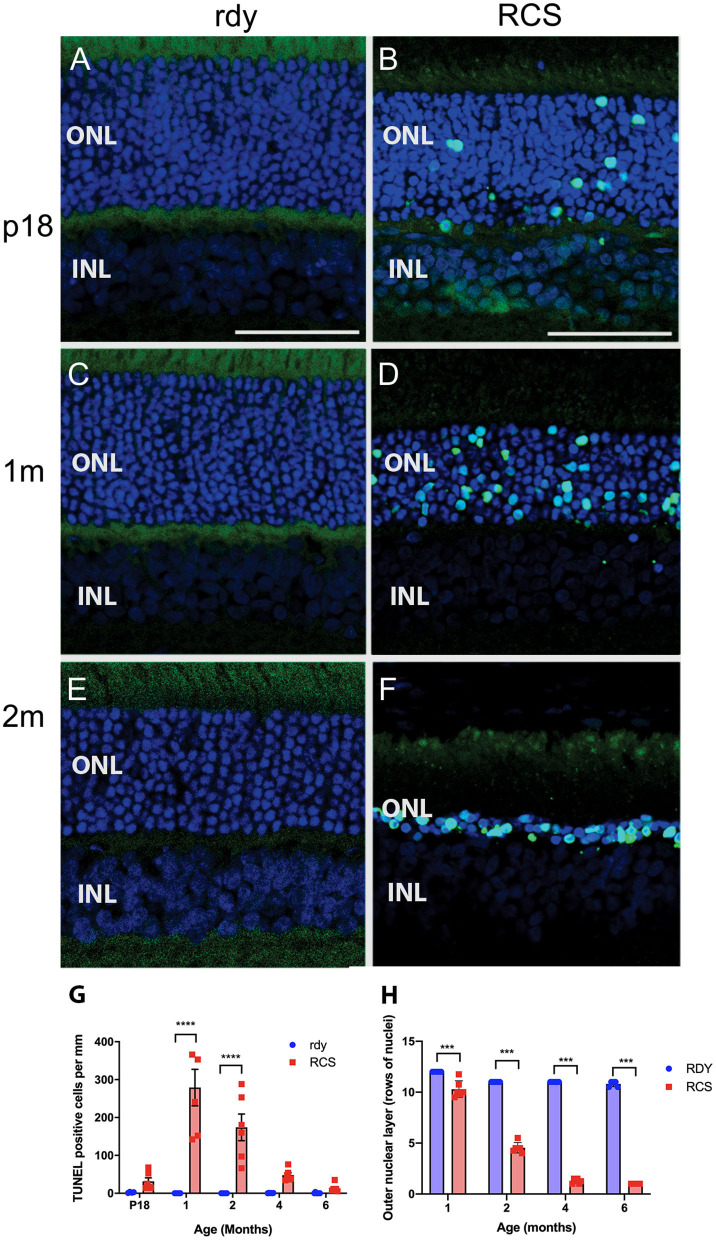
Cell death in the rdy and RCS rat retina. **(A–F)** Vertical cryostat sections of rdy **(A,C,E)** and RCS **(B,D,F)** retinae processed for TUNEL assay (green) and counterstained with nuclear stain, bisbenzimide H (blue). While no apoptotic cells were detected in rdy retinae, in RCS retinae TUNEL positive cells were detected from P18 **(B)** and increased in number at 1 **(D)** and 2 months of age. **(F)**. **(G)** Graph showing the mean ± SEM TUNEL positive cells in the rdy (unfilled bars) and RCS (filled bars) rat retina aged postnatal day 18 to 6 months of age (*n* = 4–6 rdy retinae, *n* = 6 RCS retinae). There were a significantly larger number of TUNEL positive cells in RCS rat retinae aged 1 and 2 months of age, compared to age matched control retinae (Two way ANOVA, *post-hoc* Tukeys test ****p < 0.0001 for comparisons at 1 and 2 months of age). **(H)** Graph showing mean ± SEM thickness of the outer nuclear layer (as measured by counting rows of photoreceptor nuclei) of rdy and RCS rat retinae aged 1–6 months. The outer nuclear layer was significantly thinner in the RCS rat at all ages (Two way ANOVA, ***p < 0.0001). ONL, outer nuclear layer; INL, inner nuclear layer; Scale bar: 20 μm.

Photoreceptor death was examined using a TUNEL assay (*n* = 4–6 rdy rats per age; *n* = 6 RCS rats per age). Figure 4 shows representative images of TUNEL stained rdy (Figures 4A,C,E) and RCS (Figures 4B,D,F) retinae aged postnatal day 18 (P18), and 1 and 2 months. In contrast to the control rdy retinae, there were significant numbers of labeled photoreceptor nuclei in the RCS rat retina at P18, 1 and 2 months of age, confirmed by quantifying the number of TUNEL positive cells per millimeter of retina in rdy and RCS rat retinae from P18 to 6 months of age (Figure 4G; Two way ANOVA, *post-hoc* Tukeys test, *p* < 0.0001 for comparisons at 1 and 2 months of age). A comparison of photoreceptor death in the central, mid-peripheral and peripheral retina was also investigated for all age groups, resulting in no differences between the three retinal locations (Data not shown; two way ANOVA, strain *p* = 0.0001, eccentricity *p* = 0.331; interaction *p* = 0.397). Finally, we quantified the number of rows of photoreceptor nuclei in the outer nuclear layer to quantify the change in thickness of the outer nuclear layer more accurately than is possible with OCT. As shown in Figure 4H, the outer nuclear layer is reduced in the RCS rat retina by 1 month of age, with further significant declines thereafter (Two way ANOVA *p* < 0.0001). It should be noted, however, that contrary to our observations with OCT (shown in Figure 3B), even at 6 months of age, some photoreceptor nuclei were detected and quantified using histological quantitation methods.

### Photoreceptor Loss Is More Severe in the Ventral Than the Dorsal Retina

**Figure 5:**
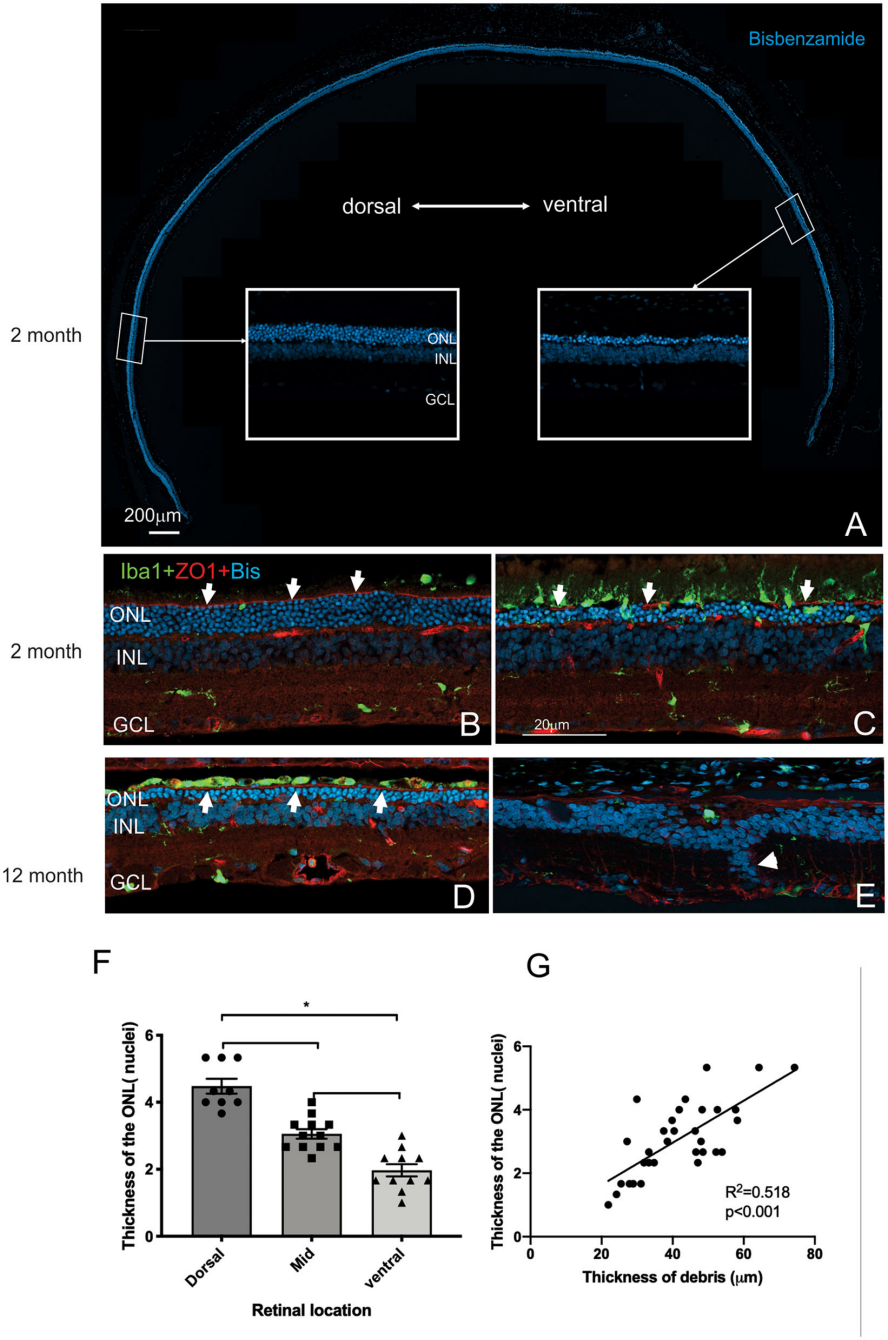
Differences in thickness of the outer retina in the dorsal and ventral retina. **(A)** Tile scan of a vertical cryostat section of RCS retina (2 months) stained with the nuclear stain bisbenzimide H in blue. Insets, displaying the dorsal and ventral retina at higher power in the regions indicated, show that the ventral outer nuclear layer (ONL) is thinner than the dorsal ONL. **(B**–**E)** Vertical cryostat sections of the dorsal **(B,D)** and ventral retina **(C,E)** stained for microglia (Iba1, green), the outer limiting membrane (ZO-1, red) and cell nuclei (BisBenzimide H, blue). **(B)** In the dorsal retina aged 2 months the outer limiting membrane (OLM, arrows, red) is continuous, whereas the ventral OLM is disrupted and fragmented (arrows, **C**) and progressively becomes more disrupted with increasing age **(E)**. However, regions of intact OLM are visible in the dorsal retina even at 12 months (**D**; arrows). The distribution of microglia cells differs between dorsal **(B)** and ventral **(C)** retina and with age, with the ventral retina showing a large number of microglia extending into the subretinal space even at 2 months of age **(C)**. **(F)** Graph showing mean ± SEM thickness of the outer nuclear layer in the dorsal, mid and ventral retina of a 2 month old RCS rats. The dorsal retina was significantly thicker than the ventral or area close to the optic nerve (mid) (One way ANOVA, *p < 0.001). **(G)** Graph showing the correlation between the thickness of the outer nuclear layer and thickness of the layer of debris within the subretinal space. In areas showing thicker debris, there were more surviving photoreceptors (Pearson's correlation, *r*^2^ = 0.518, *p* < 0.001). ONL, outer nuclear layer; INL, inner nuclear layer; GCL, ganglion cell layer. Scale bar: 20 μm.

Having established the time course of photoreceptor loss, we next evaluated histologically whether there were any differences in the rate of photoreceptor loss across the retina. Loss of photoreceptors was particularly striking in the ventral retina compared to the dorsal retina (Figure 5A). Indeed, at all ages examined the outer nuclear layer appeared thicker in the dorsal retina compared to the ventral retina (Figures 5A–E). Even in the RCS rat aged 12 months, there were regions where a thinned ONL was present in the dorsal retina (Figure 5D), whereas in the ventral retina, no photoreceptors remained, and there were signs of inner retinal remodeling with migration of nuclei into more proximal regions (Figure 5E, arrowhead). We quantified the effect of retinal location on outer nuclear layer thickness (Figure 5F). As shown in Figure 5F the number of rows of photoreceptor nuclei is greater in the dorsal retina than mid located or ventral regions (One way ANOVA, *post-hoc* Tukey's test, *p* < 0.01). In view of our previous observations that the extent of debris varies across the retina, we used histological sections to correlate the thickness of the outer nuclear layer with the thickness of debris. As shown in Figure 5G there was a significant correlation between the thickness of the debris within the subretinal space and the thickness of the outer nuclear layer (Figure 5G, Pearson's correlation, *r*^2^ = 0.518, *p* < 0.001).

Mononuclear phagocytes were scattered across the subretinal space within the regions rich in debris such as in the ventral retina (Iba-1, green; Figure 5C). In contrast, there were very few if any microglia located within the debris layer in the dorsal retina (Figure 5B). Finally, the outer limiting membrane (OLM) appeared intact in the dorsal retina (Figures 5B,D) compared to the ventral retina (see also **Figure 9A**) and corresponded to regions where the ONL appeared thicker and more organized. Notably, areas of the ventral and dorsal retina that showed an equivalent number of photoreceptor nuclei (e.g., Figures 5C,D white arrows) showed different levels of OLM integrity. In particular, OLM appeared intact in dorsal retina of the 12 month old RCS retina.

**Figure 6:**
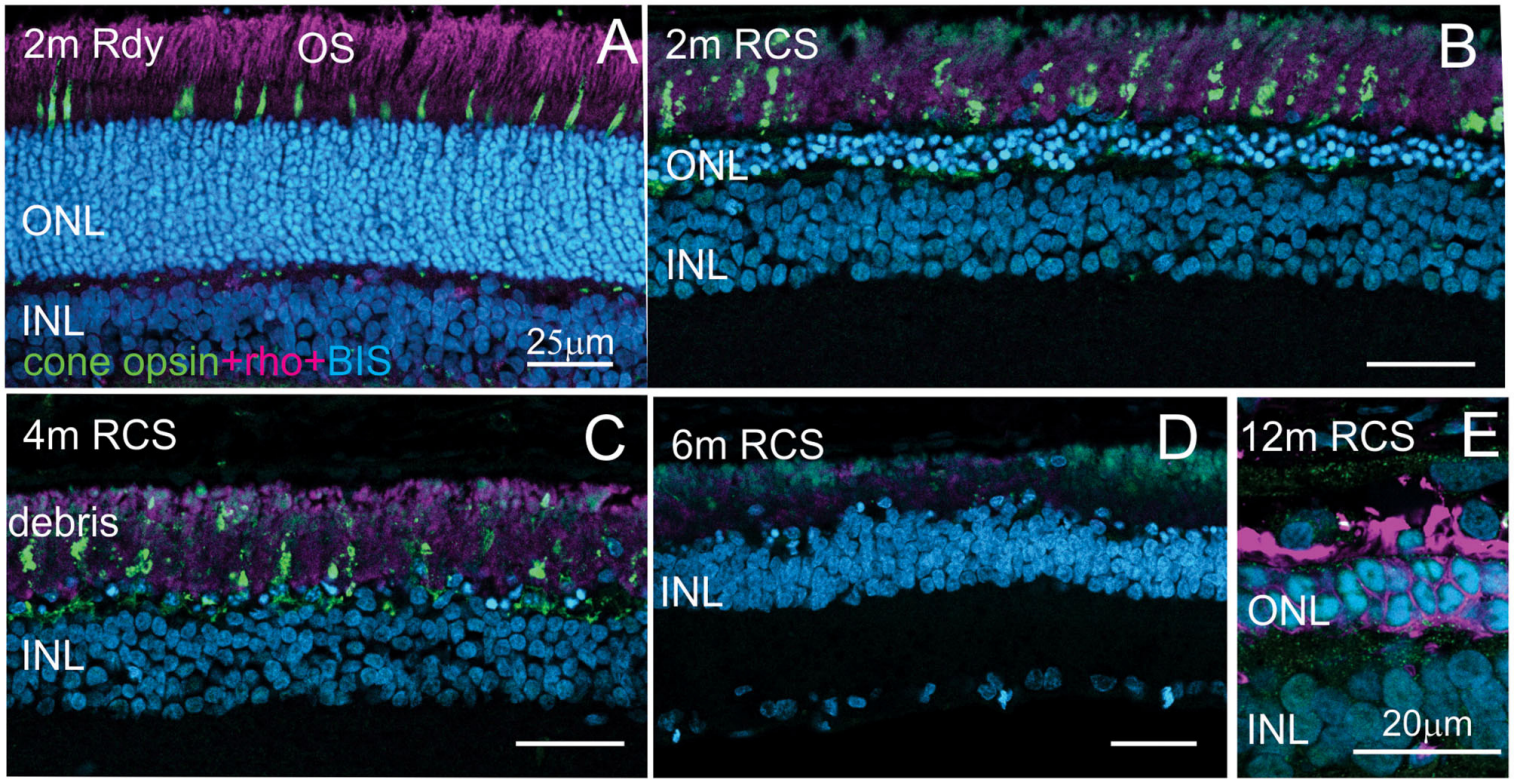
Assessment of photoreceptor outer segments in the rdy and RCS retina with age. Vertical cryostat sections of the ventral **(A–C)** and the dorsal retina **(D)** of 2, 4, 6, and 12 m old RCS retina, stained for cone outer segments (red/green opsin, green) and rod outer segments (rhodopsin, magenta). Outer segments of rod and cone outer segments are present at 2 and 4 months **(A,B)**, however, the morphology is disturbed due to the debris build-up. At 6 months **(D)** and 12 months **(E)**, outer segments have entirely disappeared. ONL, outer nuclear layer; INL, inner nuclear layer; IPL, inner plexiform layer.

Evaluation of the expression of cone-opsin and rhodopsin in the RCS rat with advancing age is shown in Figure 6 and provides an assessment of the potential function of any surviving photoreceptors. In contrast to the rdy control retina, cone- and rhodopsin labeling of photoreceptor outer segments were aberrant at 4 months and absent in the RCS retina at both 6 and 12 months of age. This implies that although photoreceptor nuclei remain in many parts of the retina with age, light sensitivity in these areas is unlikely.

**Figure 7:**
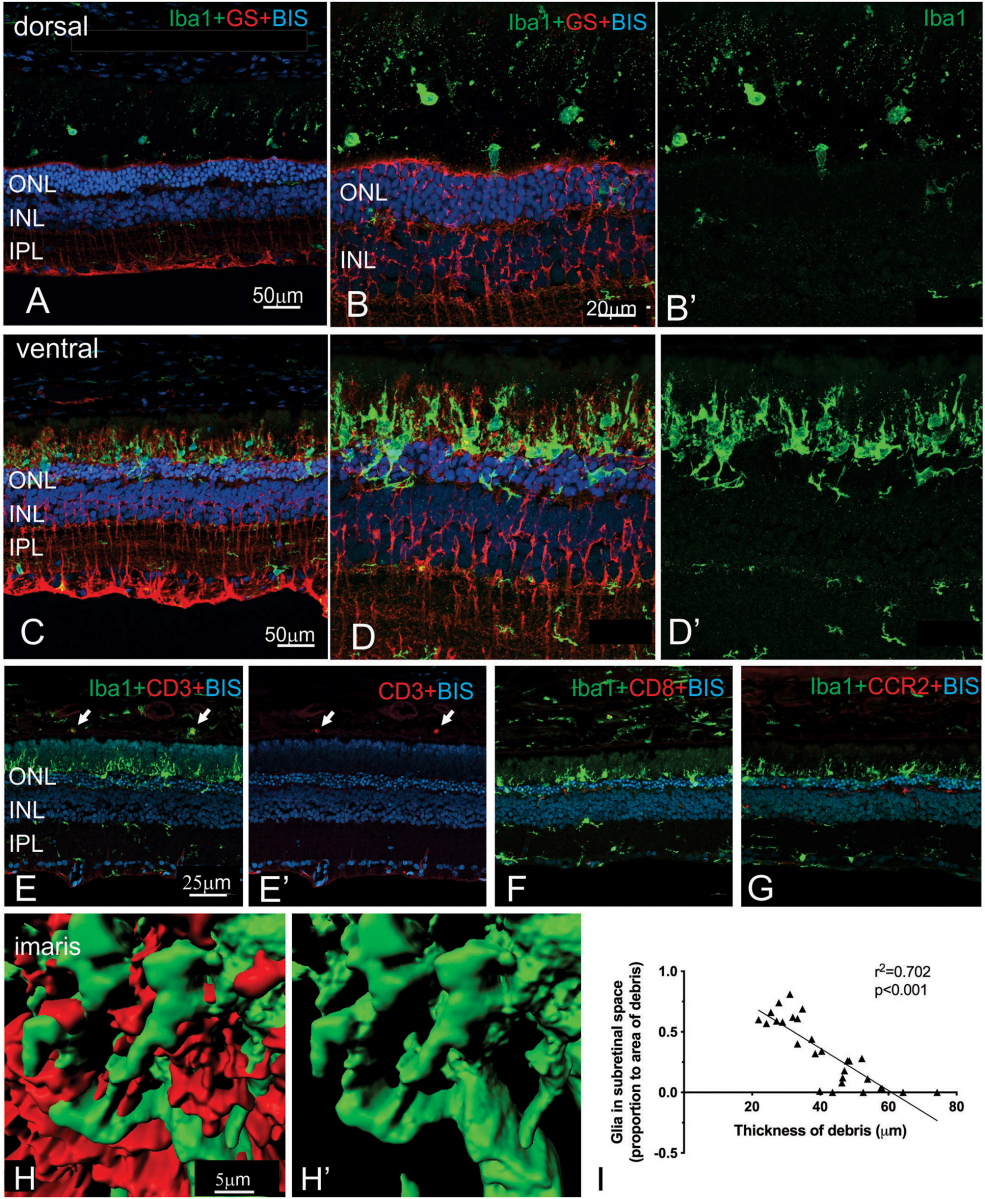
Regional differences in the morphology of microglia and Müller cells dorsal and ventral RCS retina. Vertical cryostat sections of the dorsal **(A,B,B′)** and the ventral retina **(C,D,D′)** of a 2 m RCS rat stained for microglia (Iba1, green), Müller cells (GS, red), and cell nuclei (BisBenzimide H, blue). The morphology and distribution of the microglia cells (green) is distinct between dorsal and ventral retina. Microglia in dorsal retina are distributed subretinally, scattered throughout the layer of debris away from Müller cells and have a rounded appearance. In the ventral retina Müller cells stratify into the subretinal debris area. Here, microglia cells are closely associated with the Müller cell processes and have a more ramified appearance **(C–D′,H)**. **(E,E′,F,G)** vertical cryostat sections through the ventral region of the RCS retina stained for T cell markers CD3 **(E,E′)** and CD8 **(F)**, and the chemokine receptor CCR2 **(G)**. Apart from monocytes in the choroid (arrows) and cells in blood vessels none of the antibodies stained Iba1-positive microglia or the subretinal debris region. **(H,H′)** Confocal z-stacks of the ventral, subretinal area of the 2 m RCS retina stained for microglia (Iba1, green), Müller cells (GS, red) were scanned, processed with Imaris software and represented as 3D images. Microglia cells (green) tightly co-stratify with Müller cells (red). **(I)** Graph showing a significant negative correlation between thickness of debris and amount of glia present in the subretinal space (Person's correlation; *r*^2^ = 0.7018, *p* < 0.0001). ONL, outer nuclear layer; INL, inner nuclear layer; IPL, inner plexiform layer.

More detailed examination of the microglial and Müller cell changes in the dorsal and ventral RCS retina at 2 months is shown in Figure 7. In particular, the morphology of both Müller cells and microglia was distinct in the ventral retina (Figures 7D,D′) compared to the dorsal retina (Figures 7B,B′). Notably, microglia cells were more ramified and their processes were observed extending into the subretinal space in close apposition to Müller cell processes (Figures 7C,D′,H,H′). In contrast, in the dorsal retina microglia were observed within the subretinal space, but these cells had a more rounded appearance (Figures 7A,B′). We correlated the extent of glial coverage within the subretinal space with the thickness of debris and found a highly significant negative correlation, wherein the higher the presence of glia within the subretinal space, the lower the thickness of debris (Figure 7I; Pearson's correlation *r*^2^ = 0.702, *p* < 0.0001). Colocalization of Iba1 with CD3, CD8, and CCR2 was undertaken to potentially identify any subgroups of innate immune cells within the subretinal space (Figures 7E–G). Although some cells colocalized with CD3 within the blood vessels of the choroid, no Iba1 positive cell colocalized within CD3, CD8 or CCR2 in the subretinal space.

**Figure 8:**
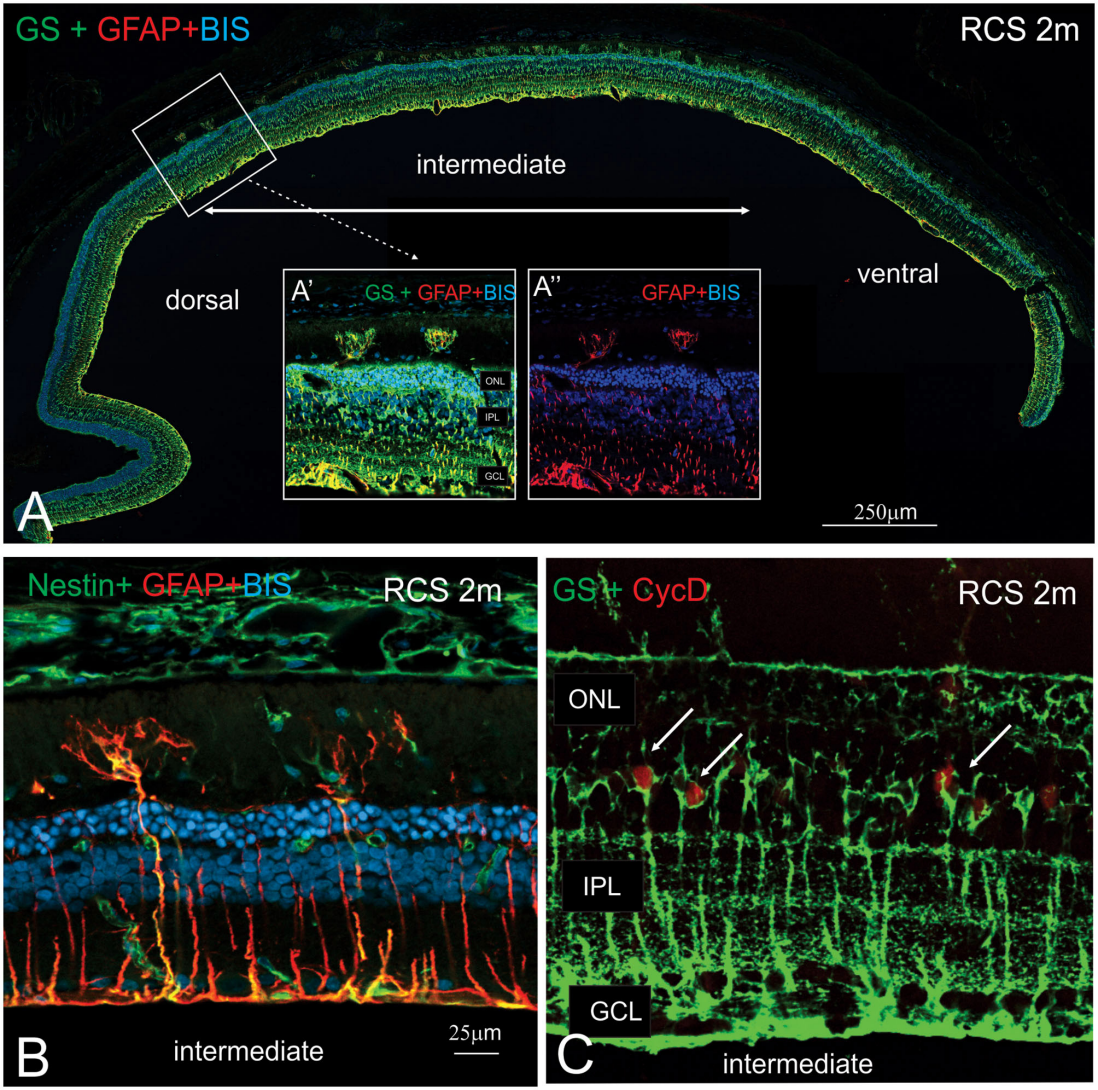
Characterization of sprouting Müller glia in intermediate region of the RCS retina. **(A)** Tile scan of a vertical cryostat section of RCS retina (2 months) stained for the intermediate filament Glial fibrillary acidic protein (GFAP, red), which labels gliotic Müller cells, glutamine synthetase, (GS, green), which labels all Müller cells and cell nuclei (BisBenzimide H, blue). Insets display area of the intermediate region of the retinal section at higher power, where two GS-GFAP positive Müller cells sprout within the subretinal space. At more ventral locations, more Müller cells processes extending into the subretinal space are apparent. **(B,C)** Vertical cryostat sections of 2 months old RCS **(B,C)** stained for Nestin and GFAP (green and red, **B**); GS and the cell cycle marker cyclin D, (green and red, **C**). At this stage of degeneration, many Müller cells appear gliotic with processes extending into the subretinal space and label for the proliferation marker, Cyclin D1. ONL, outer nuclear layer; IPL, inner plexiform layer; GCL, ganglion cell layer.

In view of the difference in thickness of the ONL between the dorsal and ventral retina, we next examined regions where the structure of the outer retina transitioned. As shown in Figure 8, Müller cells could be seen forming single sprouts within the subretinal space in a region of transition. An increasing number of Müller cells appear sprouting into the subretinal space in gradually more ventral locations (Figure 8A). These “sprouting” Muller cells were immunoreactive for the gliosis markers GFAP (Figure 8A inset A' and A”,**B**) and nestin (Figure 8B) (Valamanesh et al., 2013; Vogler et al., 2014), and also the proliferation marker cyclin D1 (Figure 8C). Overall, these findings imply that by 2 months of age, Müller cells display hallmark features of proliferative gliosis (Greferath et al., 2015).

**Figure 9:**
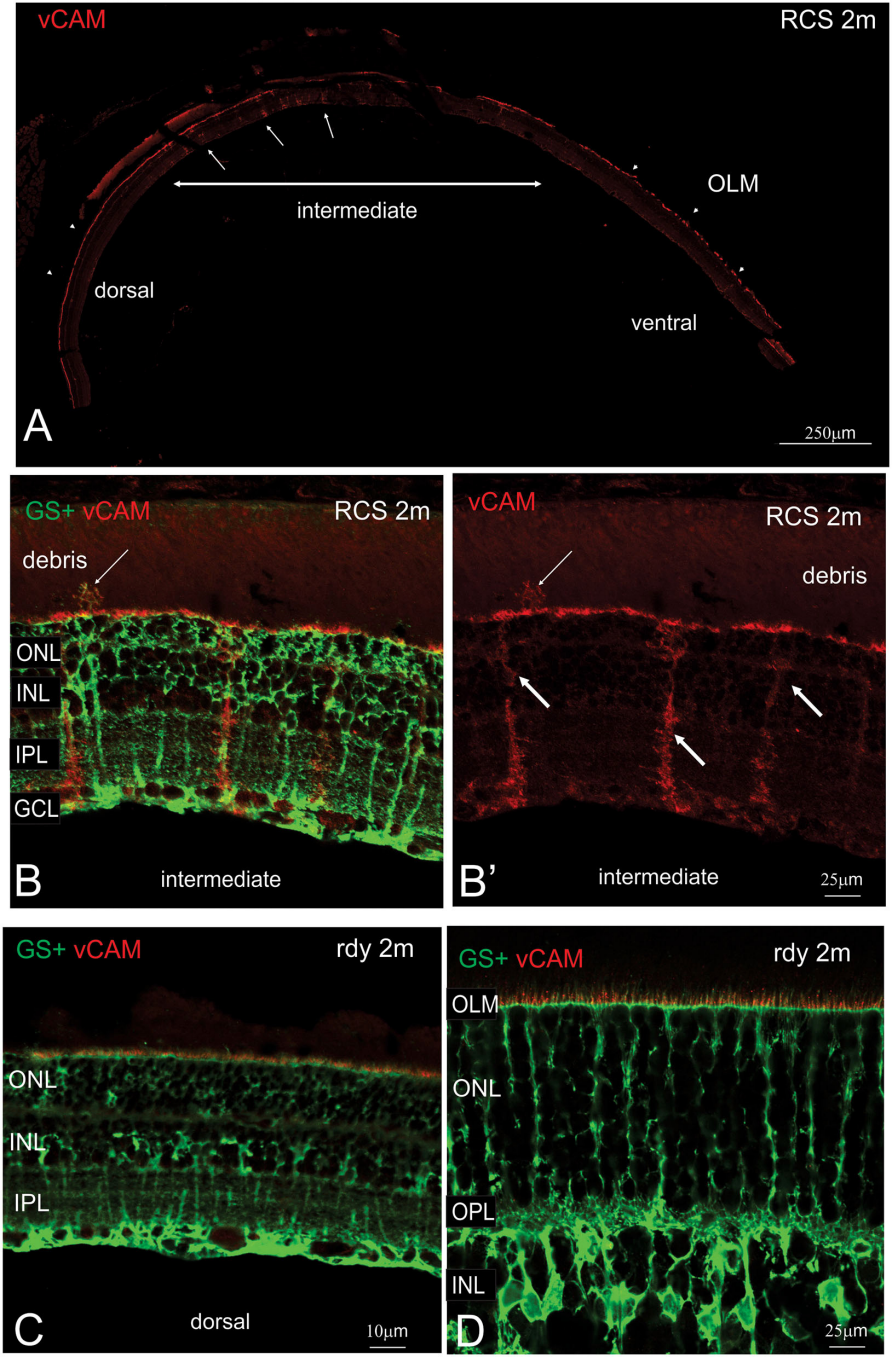
Müller cell labeling for vesicular cell adhesion molecule. **(A)** Tile scan of a vertical cryostat section of RCS retina (2 months) stained for the vesicular cell adhesion molecule (vCAM, Please note that some variability in vCAM staining is due to antigen retrieval, by which areas of the sclera/choroid detached and cover part of the retinal section). Vertical section of RCS **(B,B′)** and rdy **(C,D)** rat retinae aged 2 months immunolabelled for vCAM (red) and glutamine synthetase (green). In contrast to control rdy retinae, Müller cells in the intermediate region express vesicular cell adhesion molecule. ONL, outer nuclear layer; IPL, inner plexiform layer; GCL, ganglion cell layer.

The close apposition of mononuclear phagocytes, including possible microglia, to Müller cells in the subretinal debris area is interesting and suggests attraction and interaction between these two cell types. Wang et al. (2011) have demonstrated an upregulation of adhesion protein expression in Müller cells *in vitro* in response to microglial activation. We therefore examined vCAM expression in dorsal and ventral retinal in RCS rats aged 2 month and age-matched rdy control animals (Figure 9). In rdy retina, vCAM expression was confined to Müller cells endfeet located to a restricted region between the inner segments of the photoreceptors (Figures 9C,D). The same staining pattern was observed in the dorsal region of 2m RCS retina (Figure 9A). However, in the transition region between the dorsal and ventral retina, where Müller cells form single sprouts that extend into the subretinal space, vCAM expression was also found covering the large parts of Müller cells including the apical processes sprouting into the subretinal space (Figures 9B,B′).

## Discussion

This study characterized the long-term changes in retinal structure and function in the pigmented RCS rat in comparison to its pigmented rdy isogenic control. The major findings of this study were that although rod and cone loss occurred in the RCS rat retina from 1 month of age, the rate of photoreceptor loss was particularly pronounced in the ventral retina compared to the dorsal retina. Moreover, the level of photoreceptor loss was inversely correlated with the level of debris within the subretinal space, disruption of the outer limiting membrane, and a significant change in mononuclear phagocytes and gliotic Müller cells within the subretinal space.

The death rate of rods and cones has been well-characterized in a number of animal models of retinal degeneration. Where causative mutations affect rod specific proteins, rods generally die prior to cone death (Punzo et al., 2009). Our results demonstrate that rod mediated function was more severely affected at an early age than cone function, a finding that is similar to the mer^kd^ mouse, a mouse that has a targeted deletion in merTK (Duncan et al., 2003). The mutation causing retinal degeneration in the RCS rat is in merTK (Vollrath et al., 2001), a critical tyrosine kinase involved in engulfment of both rods and cone outer segments by the RPE. It is not clear why there is more rapid degeneration of neurons in the rod compared to cone pathway. Although merTK is important for phagocytosis of both rods and cones, other components that mediate phagocytosis may be distinct in rods or cones and could potentially lead to a difference in rate of degeneration of cones or rod photoreceptors. In addition, loss of the RPE is associated with a lower level of cone disruption than rods, as observed in transgenic RPE^CreER^/DTA transgenic mice that have sporadic loss of RPE cells (Busskamp et al., 2008). This suggests that although the RPE is important for maintaining both rods and cone integrity, cones are also likely to depend on other non-RPE factors.

One of the key findings in this study was the observed differences in photoreceptor death between the dorsal and ventral retina. The differences noted were clearly shown using immunohistochemical evaluation of vertical retinal sections that included all retinal eccentricities. However, differences between the superior and inferior retina were also evident in OCT scans. Differences in the photoreceptor integrity between the dorsal and ventral retina has been implied previously in studies examining the inner retinal vascular pathology in the RCS rat. Although not quantified, both Shen et al. (2014) and Zambarakji et al. (2006) show images where vascular anomalies were more prevalent in the inferior retina than the superior retina of the RCS rat. In addition, the rate of loss in density of cone inner segments has been shown to be greater in the inferior retina than superior retina (Huang et al., 2011). Two recent studies using OCT imaging, failed to report variation in outer retinal thickness in the dorsal and ventral retina (Adachi et al., 2016; Ryals et al., 2017). It is possible that those studies methods used to quantify individual retinal layers, and especially the outer retina, were not sensitive enough to detect the variations in extent of debris and photoreceptors in the different locations.

Preferential loss of photoreceptors in the inferior retina is a feature of many forms of sectorial retinitis pigmentosa, as well as retinal degeneration caused by toxic ablation of the RPE such as by sodium iodate. We considered whether the differences in photoreceptor loss in the dorsal and ventral retina could be explained by the differential effects of light exposure. Indeed, Zhao et al. suggested that photoreceptor death in mertk^−/−^ mice could be explained by light induced toxicity mediated by bisretinoids (Zhao et al., 2018), which would be more highly concentrated in areas with greater levels of outer segment debris. However, light induced oxidative stress is known to cause rapid loss of photoreceptors in the dorsal retina, rather than the ventral retina–the opposite to that was observed in this study (Tanito et al., 2008).

The mechanism(s) of photoreceptor death in the RCS rat retina are not well-understood. Previous studies have suggested that photoreceptor death occurs in the RCS rat as a consequence of the build-up of debris within the subretinal space (Dowling and Sidman, 1962). The debris, which is rich in spent photoreceptor outer segments, especially bisretinoids, may either exert a toxic effect on photoreceptors (Zhao et al., 2018), or alternatively, act as a barrier to the diffusion of vitamins, especially retinoids, and other vital nutrients to and from the choroidal vasculature. However, our observation that the level of debris was strikingly different in the dorsal and ventral retina contradicts this notion. Notably, we observed a greater level of debris in the dorsal retina, a region that coincided with a greater number of surviving photoreceptors. With a higher number of surviving photoreceptors in the dorsal retina, the level of debris in the dorsal retina may be a reflection of the number of photoreceptors requiring outer segment turnover.

A striking difference in the structure of the dorsal retina compared to the ventral retina was the integrity of the outer limiting membrane and also the number and location of innate immune cells, including microglia. Importantly, the outer limiting membrane was intact in the dorsal retina and showed very few mononuclear phagocytes within the subretinal space, whereas the converse was observed in the ventral retinal. This observation may be explained in two different ways. The most obvious explanation, for the difference in the OLM in the dorsal and ventral retina is a difference in rate of degeneration in the two regions. Indeed, Hippert et al. (2015) showed disruption of the OLM late in the course of degeneration in a Rho-/- mouse model of retinal degeneration. Alternatively, there may be changes in expression of proteins critical for maintaining the OLM that subsequently lead to or exacerbate photoreceptor degeneration. The outer limiting membrane contains a range of adhesion proteins, that form a complex allowing adhesion between Müller cells and the inner segment of photoreceptors (Mehalow et al., 2003). Localized loss of photoreceptor polarity and subsequent degeneration occurs, when one or more of these outer limiting membrane protein are disrupted, as is seen in the Crb1^rd8/rd8^ model of retinal degeneration (van de Pavert et al., 2004; Hippert et al., 2015). In addition, the outer limiting membrane of the *rd1* mouse model is known to be disrupted across both the superior and inferior retina from an early stage of degeneration (Hippert et al., 2015). In this study, the OLM remained intact in the dorsal RCS retina until an advanced age. Indeed, pockets of surviving photoreceptor were present in the dorsal retina in regions where the OLM remained intact, suggesting that changes in OLM integrity could contribute to photoreceptor loss in the ventral retina. An additional implication of the localized breakdown of the OLM is the migration of RPE cells and associated pigment that is known to be a feature of end stage retinitis pigmentosa. More work is needed to establish the role of outer limiting membrane integrity in exacerbation of photoreceptor death during retinal degeneration.

A similar argument could be made for the difference in microglia morphology and interaction with Müller processes that was observed in the ventral retina. With more advanced disease, and greater levels of photoreceptor death, more microglia would be expected to accumulate within the subretinal space. Indeed, several studies have shown increasing numbers of Iba1 positive cells (likely microglia or subretinal macrophages) in the subretinal space from postnatal 14 (He et al., 2019; Lew et al., 2020). However, comparison of dorsal and ventral retinae with equivalent levels of surviving photoreceptors (i.e., 12 month old dorsal retina compared with 2 month old ventral retina), shows that the ventral retina has a disproportionate number of microglia for the equivalent photoreceptor death. In addition, the increase in vCAM expression by Müller cell processes, suggests a potential mechanism by which microglia or other mononuclear phagocytes could adhere to Müller cell processes within the subretinal space. Extension of Müller cell processes into the subretinal space has been previously shown (Lassiale et al., 2016). What has not been considered previously is whether there is an association between these processes and microglia that potentially migrate into the subretinal space in response to photoreceptor death. Microglia and other mononuclear phagocytes are known to release a range of inflammatory cytokines, and their sheer number could exert cytotoxic effects on neighboring photoreceptors. Indeed, a recent study showed increased expression of the chemokine Ccl5 from postnatal 14 in the RCS rat retina and suppression of microglia activity with tamoxifen or the liposome clodronate reduced photoreceptor death (Lew et al., 2020). Moreover, there is some evidence that microglia migrate into the subretinal space of the RCS or mertk-/- mouse in response to photoreceptor death (Kohno et al., 2015; Lew et al., 2020). When taken together, we propose that breakdown in the outer limiting membrane in the ventral retina is critical for the extension of glial processes and migration of microglia into the subretinal space, that ultimately exacerbates photoreceptor death via release of cytokines (e.g., Ccl5) in this region of the retina.

In conclusion, this study shows that retinal degeneration occurs in a non-uniform fashion across the retina of the RCS rat, with the ventral retina more profoundly affected than the dorsal retina. Importantly, the level of debris was lower in the ventral retina, the number of photoreceptors reduced, whilst mononuclear phagocytes and penetrating gliotic Müller cell processes were more abundant. These findings are significant for several reasons. First, studies evaluating the efficacy of treatments need to be controlled carefully for retinal location, so as to take into account the differences in photoreceptor integrity in the dorsal and ventral retina. Secondly, these results highlight the value of using the RCS rat model as a model to examine sectorial RP. Finally, further work is needed to explore the potential contributing role that breakdown of the outer limiting membrane, protrusion of Müller cells and migration of microglia and other mononuclear phagocytes have in exacerbating photoreceptor death in this and other retinal degenerations.

## Data Availability Statement

The raw data supporting the conclusions of this article will be made available by the authors, without undue reservation.

## Ethics Statement

The animal study was reviewed and approved by The University of Melbourne Animal Experimentation Ethics Committee.

## Author Contributions

UG and MH contributed equally to the study design, data collection, data analysis, and writing. GV collected data and undertook data analysis and contributed to writing the manuscript. AJ and KV undertook data analysis and contributed to writing the manuscript. DS, HO'N, and IL contributed to writing the manuscript and analyzing data. EF undertook data analysis, wrote the manuscript, and supervised the project. All authors contributed to the article and approved the submitted version.

## Conflict of Interest

The authors declare that the research was conducted in the absence of any commercial or financial relationships that could be construed as a potential conflict of interest.
